# Antibiotic prescribing habits of the clinicians dealing with dental implant surgery in Turkey: a questionnaire study

**DOI:** 10.1186/s40729-020-00252-4

**Published:** 2020-09-27

**Authors:** Gül Merve Yalcin-Ülker, Merve Cakir, Deniz Gökce Meral

**Affiliations:** 1 Faculty of Dentistry, Oral and Maxillofacial Surgery Department, Istanbul Okan University, Aydıntepe, Tuzla, 34947 Istanbul, Turkey; 2 Professor, Head of the Department, Oral and Maxillofacial Surgery Department, Istanbul Okan University, Aydıntepe, Tuzla, 34947 Istanbul, Turkey

**Keywords:** Antibiotic prescription, Implant dentistry, Postoperative, Preoperative, Prophylaxis, Questionnaire study

## Abstract

**Purpose:**

Although various prophylactic and/or postoperative systemic antibiotic regimens have been suggested to minimize failure after dental implant placement and postoperative infection, the role of antibiotics in implant dentistry is still controversial. The purposes of this questionnaire study were to determine the current antibiotic prescribing habits of clinicians in conjunction with dental implant placement and to understand whether any consensus has been reached among implant surgery performing clinicians.

**Methods:**

An electronic questionnaire was sent by electronic mail to all members of the Turkish Dental Society. The questions were related to whether antibiotics were routinely prescribed either pre- or/and postoperatively during routine dental implant placement. The respondents were also asked to specify their workplace and education. The results were analyzed using SPSS software. Descriptive and chi-square analyses were used to compare categorical data; Kruskal–Wallis test was used to compare the quantitative data by category.

**Results:**

A total of 429 members responded to the questionnaire. The clinicians having more experience had a greater tendency to prescribe preoperative antibiotics (*p* < 0.001), but there was no statistically significant difference between the postoperative antibiotic prescription choice of the clinician according to the clinicians’ experience (*p* > 0.05). A total of 175 of the clinicians preferred to prescribe preoperative antibiotics when there was systemic comorbidity; 99 of the clinicians preferred to prescribe antibiotics before every implant surgery. The aminopenicillins were the most commonly prescribed antibiotics by the clinicians. A total of 38.58% of the respondents (*n* = 130) who were prescribing preoperative antibiotics, 2000 mg aminopenicillin was given 1 h before the surgical procedure. Dentists and solo private practitioners were prescribing more preoperative antibiotics (*p* < 0,05).

**Conclusions:**

There was no consensus among clinicians regarding the use of antibiotics in association with routine dental implant placement. Aminopenicillins were the most commonly prescribed antibiotics for both pre- and postoperatively. Furthermore, most of the antibiotic regimens being used are not in accordance with the current published data.

## Introduction

The basic aim of the dentistry is to protect and/or restore dentomaxillofacial structures. Especially, coping with missing teeth of the patients is one of the main duties of dental professionals. Nowadays, the best promising routine way to replace a missing or poorly prognosed tooth with high success and survival rates is placing dental implants [1]. Considering that dental implant placement is a routine surgical treatment in the dental offices, it is necessary to understand the factors affecting implant success and survival. There are many factors affecting implant success and survival. Foremost, among these factors is early and late implant site infection [2]. Considering that dental implant treatment is now a routine surgical treatment, it is necessary to understand the effect of antibiotics on the success of such a frequently performed procedure.

During placement of dental implants, oral microflora should be controlled, as pathogenic bacteria present in the oral environment may cause peri-implant infections in implants as well as in the teeth, which may impair the early healing of the implant, i.e., osseointegration [3, 4]. Unfortunately, there are still controversial results regarding perioperative antibiotic use and many of the current clinical data have the risk of bias [5]. Many different pre- and postoperative antibiotic regimens have been proposed to prevent postoperative infection and indirect early implant loss. Adell et al. recommended 2 g penicillin-V (phenoxymethylpenicillin) orally 1 h before the procedure and advised to continue after the procedure for 10 days twice daily [6]. In more recent protocols, 2 g of penicillin-V orally (or amoxicillin or amoxicillin + clavulanate) 1 h before the procedure was recommended, followed by 500 mg of penicillin-V 4 times a day in the postoperative period [7]. In addition, systematic reviews and meta-analyses of dental implant treatment are available in the literature. In a Cochrane systematic review by Esposito et al., which included six randomized clinical trials (RCTs) in 2013, it was reported that amoxicillin administered 1 h before dental implant surgery is effective in implant site infection in the postoperative period, but there was no conclusion about postoperative antibiotic use [8]. In a meta-analysis conducted by Braun et al., including eight RCTs in 2019, it was determined that preoperative antibiotic use may reduce postoperative infection when the clinical studies in the literature were evaluated, but the available information in the literature cannot be a complete result due to the heterogeneity in the studies [9]. They argued that postoperative or pre- and postoperative antibiotic use cannot be explained by the available scientific data [9].

Also, a recent network meta-analysis (NMA) by Romandini et al. stated that data in the current literature is unable to reach high evidence [5]. As a result of systematic reviews and meta-analyses conducted to date, it has been demonstrated that preoperative antibiotic use (2 g or 3 g orally 1 h before the procedure) before intrabony dental implants in healthy individuals plays a role in reducing postoperative infection-induced implant failures [8, 10]. However, as Romandini et al. stated in their recent NMA, a single preoperative dose of 2 g of amoxicillin does not appear to be indicated with high evidence in the current literature [5]. Furthermore, a single preoperative dose of 3 g of amoxicillin regimen was evaluated in one single RCT with a high failure rate in the control group [10]. Being one single trial and suggesting a high dose of amoxicillin considering its side effects make it necessary to think about this regimen.

The use of postoperative antibiotics is also a controversial situation in the literature. Due to heterogeneity and insufficient data in clinical studies of postoperative antibiotic use in the literature, there is no conclusive report regarding its relationship with postoperative infection [8].

Clinicians should never forget that the extensive use of antibiotics in order to decrease implant failures could bring its disadvantages. It should not be considered that antibiotic administration on patients may be related only to dental implant survival or success. Improper antibiotic use also has both individual and social effects. Hypersensitivity reactions caused by antibiotics can cause serious medical problems [11]. In addition, the use of some antibiotics may cause gastrointestinal side effects by causing opportunistic infections in a considerable amount of individuals in the community [12]. Among these effects, the most serious problem in the long term is the resistance developed by bacteria against antibiotics and the superinfections that these bacteria can cause. Epidemiological studies have shown a direct correlation between antibiotic consumption and the emergence of resistant bacterial strains [13]. For these reasons, considering the consequences, antibiotics should not be prescribed indiscriminately. Before prescribing antibiotics, clinicians should rethink that with its whole consequences. In 2015, European Association for Osseointegration (EAO) stated in its consensus report that in straightforward cases, antibiotic prophylaxis has not shown a beneficial effect; but in complex cases like procedures with grafting or immediate placement of implants and/or patients with systemic comorbidities, antibiotic prophylaxis has a beneficial effect [14]. Another systematic review by Lund et al. stated that preoperative antibiotic use may reduce the risk of implant failure, but the subanalysis of the primary studies showed that it is unnecessary to prescribe preoperative antibiotics before uncomplicated implant surgeries in healthy patients [15].

However, the fact that in the literature has not been reached regarding pre- and/or postoperative antibiotic use related to implant surgery, the conclusion in the literature means that clinicians lack the full scientific resources available to them for making their clinical decisions.

The aim of this study was to determine the pre- and/or postoperative antibiotic prescription habits of clinicians in Turkey who have been performing dental implant surgery according to their year of experience, presence of specialty in the field, their title, and workplace. In this study, the circumstances in which the clinicians prefer prescribing preoperative antibiotics were also investigated, so as to understand whether there is a conclusion among clinicians, and to determine the level of compliance of the clinicians' habits with the current literature.

## Materials and method

This study was designed as a cross-sectional questionnaire intending to understand antibiotic prescribing habits of the clinicians performing implant surgery in Turkey. Ethical approval was obtained from the Istanbul Okan University Ethical Committee (Istanbul, Turkey) with the acceptance protocol 98. An electronic questionnaire was created with Google® Forms (Google®, California, USA). The questionnaire link was sent to all those of the members of Turkish Dental Association (Türk Dişhekimleri Birliği [TDB]) via e-mail by the secretary service of the association and the dynamic responses were automatically recorded on to the Google® Forms for 3 months (February–April 2019). All responses were anonymous. The questionnaire consisted of 15 questions. The clinicians were asked to assume that the patients had no antibiotic allergies. Generally, there were three subdivisions in the questionnaire, the qualifications of the clinician, preoperative antibiotic prescription, and postoperative antibiotic prescription habits (Tables 1, 2, and 3). In the first part, the experience of the clinician as a dental clinician and also as an implant surgery-performing clinician; the workplace of the clinician (solo private practice, group private practice, oral dental health centre of the state, or university clinic) and if there were any, implant surgery-related specialties (oral and maxillofacial surgeon, periodontologist, oral implantologist) of the clinicians were also recorded. After this section, the incidence of preoperative and postoperative antibiotic prescription related to implant surgery was recorded including the type, the dosage, and the duration. The questions about the antibiotic regimen were translated into Turkish language by two experienced bilingual oral and maxillofacial surgeons from the questionnaire by Debb et al. and modified for Turkish clinicians [16]. In addition to these questions, the reasons for preoperative antibiotic prescription were also recorded. According to the design of the questionnaire, one could choose only one antibiotic regimen for each of the pre- and postoperative sections. Finally, because of the irrelevancy of this parameter according to the authors of this study, there was no any defining question about the gender of the clinicians.

**Table 1:** Questions regarding qualifications of the clinician

1. How many years of experience do you have in dental practice?	
2. You are:	Dentist
	Specialist in implant surgery-related branches (oral and maxillofacial surgeon, periodontologist, oral implantologist)
	Postgraduate student in implant surgery-related branches (oral and maxillofacial surgeon, periodontologist, oral implantologist)
3. Where do you work?	Orodental Health Centre of the State
	University Clinic
	Solo private practice
	Group private practice
4. How many years of experience do you have in implant surgery?

**Table 2:** Questions about PREoperative antibiotic prescription habits of the clinicians

5	Do you prescribe PREoperative antibiotics before implant surgery?		Sometimes	
			Yes	
			No	
6	In which circumstances do you prescribe preoperative antibiotics?		Multiple-implant surgery	
			Every implant surgery	
			Smoking patients	
			Patients with systemic comorbidities (diabetes mellitus, steroid usage, patients with immunosuppression)	
7	When do you start preoperative antibiotics prior to implant placement?		__ Hour(s) or __ Day(s)	
8	Which antibiotic do you prefer? (with Turkish Brand Names)—Please assume that the patients had no antibiotic allergies.		Aminopenicillins, Aminopenicillins+Betalactamase Inhibitors	
			Clindamycin	
			Spiramycin	
			Cephalosporins	
			Erythromycin	
			Clarithromycin	
			Antianaerobics (Metronidazol, Ornidazol)	
9	if	Aminopenicillins, Aminopenicillins+Betalactamase Inhibitors	__ 500 mg __ 625 mg __ 1000 mg or __ 2000 mg	__ Once __ BID __ TID __ QID
		Clindamycin	150 mg__300 mg__600 mg	__ Once __ BID __ TID __ QID
		Spiramycin	3__MIU	__ Once __ BID __ TID __ QID
		Cephalosporins	500 mg__1000 mg__2000 mg	__ Once __ BID __ TID __ QID
		Erythromycin	250 mg__500 mg	__ Once __ BID __ TID __ QID
		Clarithromycin	250 mg__500 mg	__ Once __ BID __ TID __ QID
		Antianaerobics (Metronidazol, Ornidazol)	250 mg__500 mg	__ Once __ BID __ TID __ QID
10	Preferred Route of Administration		Oral	
			Parenteral (IM/IV)	

*Abbreviations*: *IM* intramuscular, *IV* intravenous, *BID* twice daily, *TID* 3 times daily, *QID* 4 times daily

**Table 3:** Questions about POSToperative antibiotic prescription habits of the clinicians

11	Do you prescribe POSToperative antibiotics?		Sometimes	
			Yes	
			No	
12	Which antibiotic do you prefer? (with Turkish Brand Names)—Please assume that the patients had no antibiotic allergies.		Aminopenicillins, Aminopenicillins+Betalactamase Inhibitors	
			Clindamycin	
			Spiramycin	
			Cephalosporins	
			Erythromycin	
			Clarithromycin	
			Antianaerobics (Metronidazol, Ornidazol)	
13	if	Aminopenicillins, Aminopenicillins+Betalactamase Inhibitors	500 mg__625 mg__1000 mg__2000 mg	__ Once __ BID __ TID __ QID
		Clindamycin	150 mg__300 mg__600 mg	__ Once __ BID __ TID __ QID
		Spiramycin	3__MIU	__ Once __ BID __ TID __ QID
		Cephalosporins	500 mg__1000 mg__2000 mg	__ Once __ BID __ TID __ QID
		Erythromycin	250 mg__500 mg	__ Once __ BID __ TID __ QID
		Clarithromycin	250 mg__500 mg	__ Once __ BID __ TID __ QID
		Antianaerobics (Metronidazol, Ornidazol)	250 mg__500 mg	__ Once __ BID __ TID __ QID
14	Prefered route of administration		Oral	
			Parenteral (IM/IV)	
15	Duration (days)			

*Abbreviations*: *IM* intramuscular, *IV* intravenous, *BID* twice daily, *TID* 3 times daily, *QID* 4 times daily

The data was analyzed with Statistical Package for Social Sciences (SPSS) for Windows Version 23 (SPSS V23; IBM® Corporation, Armonk, New York, U.S.A). The descriptive and chi-square analyses were used to compare the categorical data. The Kruskal-Wallis test was used to compare the quantitative data by category. The analysis results were expressed as median values (minimum-maximum) for the quantitative data and frequency (percentage) for the categorical data. The significance level was taken as *p* < 0.05.

## Results

A total of 429 of the 19,963 (2.15%) members of the TDB responded to the questionnaire. The preference of the preoperative antibiotic prescription habit according to the years of experience of the clinicians is shown in Table 4. For both experience parameters, the median values showed a statistically significant difference (*p* < 0.001). The clinicians having more experience had a greater tendency to prescribe preoperative antibiotics.

**Table 4:** The relationship between experience years and preoperative antibiotic prescription habit

	Experience years		Experience in implant surgery	
	Median (min.-max.)	*n*	Median (min.-max.)	*n*
Sometimes	10 (1-45)^a^	194	6 (0-31)^a^	183
Yes	22 (1-53)^b^	143	10 (1-30)^b^	136
No	11,5 (1-42)^a^	92	5 (0-29)^a^	12
*p*	**<0,001**		**<0,001**	

^a-b^There were no statistically significant differences between median values with the same letters

In Table 5, the preference of postoperative antibiotic prescription according to the experience of the clinician showed that there was no statistically significant difference for the postoperative antibiotic prescription choice of the clinician according to the clinicians’ experience (*p* > 0.05).

**Table 5:** The relationship between experience years and postoperative antibiotic prescription habit

	Experience years		Experience in implant surgery	
	Median (min.-max.)	*n*	Median (min.-max.)	*n*
Sometimes	17 (1-42)	60	2 (1-120)	55
Yes	12 (1-53)	342	6 (1-120)	255
No	19 (1-44)	22	7,5 (1-48)	18
*p*	0,094		0,868	

When the preoperative antibiotic usage preferences (every implant surgery, multi-implant surgery, patients who smoke, and patients with systemic comorbidities) were compared, a significant difference was observed between the parameters (*p* < 0.001) (Table 6). A total of 91.6% of the clinicians prescribing preoperative antibiotics ‘sometimes’ preferred preoperative antibiotics in the cases of patients with systemic comorbidities.

**Table 6:** The circumstances in which clinicians prefer to prescribe preoperative antibiotics

	Multiple-implant surgery *n*(%)	Every implant surgery *n*(%)	Smoking patients *n*(%)	Patients with systemic comorbidities *n*(%)	*p*
Sometimes	14 (7.3)	0 (0)	2 (1)	175 (91.6)*	**<0.001**
Yes	16 (11.2)	99 (69.2)	0 (0)	28 (19.6)	
No	2 (10.5)	0 (0)	0 (0)	17 (89.5)	

*Statistically significant differences

The analysis of the answers given to the question ‘Do you prescribe PREoperative antibiotics?’ according to the title (dentist, specialist, or postgraduate student) showed a statistically significant difference (*p* = 0.002) (Table 7). While 39.4% of dentists stated that they were prescribing preoperative antibiotics sometimes and 39.1% of the dentists prescribed preoperative antibiotics in every case, 63.2% of the postgraduate students and 54.2% of the specialists stated that they are sometimes prescribed preoperative antibiotics.

**Table 7:** The relationship between antibiotic prescribing habits and the titles of the clinicians.

	Dentist *n*(%)	Postgraduate student *n*(%)	Specialist *n*(%)	*p*
Do you prescribe PREoperative antibiotics?				
Sometimes	112 (39.4)	24 (63.2)	58 (54.2)	**0.002**
Yes	111 (39.1)	9 (23.7)	23 (21.5)	
No	61 (21.5)	5 (13.2)	26 (24.3)	
Do you prescribe POSToperative antibiotics?				
Sometimes	42 (15.1)	3 (7.9)	15 (14)	0.671
Yes	221 (79.2)	34 (89.5)	87 (81.3)	
No	16 (5.7)	1 (2.6)	5 (4.7)	
In which cases do you prefer to prescribe PREoperative antibiotics?				
Multiple-implant surgery	25 (10.8)	3 (8.6)	4 (4.8)	**0.014**
Every implant surgery	76 (32.7)	5 (14.3)	18 (21.4)	
Patients with systemic comorbidities	131 (56.5)	27 (77.1)	62 (73.8)	

There was a statistically significant difference between the choices of the clinicians according to their titles (*p* = 0.014) (Table 7). The presence of systemic co-morbidity affects clinicians’ decision in the prescribing of antibiotics preoperatively. While 56.2% of the dentists stated that they were prescribing preoperative antibiotics when there was a systemic comorbidity, 77.1% of the postgraduate students and 73.8% of the specialists were prescribing preoperative antibiotics in this case.

There was no statistically significant difference in the postoperative antibiotic prescribing habit according to the titles of the clinicians (*p* = 0.671) (Table 7).

In Tables 8 and 9, the choice of the antibiotic regimen of the clinicians was shown. Pre- and postoperatively, the aminopenicillins were the most commonly prescribed antibiotics by the clinicians. For 38.58% of the respondents (*n* = 130) who were prescribing preoperative antibiotics, 2000 mg aminopenicillin given 1 h before the surgical procedure was the clinical choice followed by 19.3% of the respondents (*n* = 65) who preferred 1000 mg aminopenicillin given 2 times per day before 1 day of the procedure and aminopenicillin given 2 times per day before 2 days of the procedure 17.5% of the respondents (*n* = 59). Postoperatively, the most common antibiotic regimen was 1000 mg aminopenicillin two times per day for 5 days (53.5%).

**Table 8:** Preoperative antibiotics preferred by the clinicians with regimen and dosage

Before:	Antibiotic	Dose	***n***
**Half an hour**	Aminopenicillins, Aminopenicillins+Betalactamase Inhibitors	2000 mg	3
**1 h**	Aminopenicillins, Aminopenicillins+Betalactamase Inhibitors	2000 mg	130
	Clindamycin	600 mg	2
	Clarithromycin	500 mg	1
	Cephalosporins (IM or IV)	2000 mg	9
**2 h**	Aminopenicillins, Aminopenicillins+Betalactamase Inhibitors	2000 mg	26
	Clindamycin	600 mg	1
**3 h**	Clindamycin	600 mg	1
**6 h**	Aminopenicillins, Aminopenicillins+Betalactamase Inhibitors	2000 mg	3
**8 h**	Aminopenicillins, Aminopenicillins+Betalactamase Inhibitors	2000 mg	1
**12 h**	Aminopenicillins, Aminopenicillins+Betalactamase Inhibitors	2000 mg	6
**1 day**	Aminopenicillins, Aminopenicillins+Betalactamase Inhibitors	1000 mg	5
	Aminopenicillins, Aminopenicillins+Betalactamase Inhibitors	1000 mg BID	65
	Antianaerobics (Metronidazol, Ornidazol)	500 mg TID	1
	Spiramycin	3 MIU BID	1
**2 days**	Aminopenicillins, Aminopenicillins+Betalactamase Inhibitors	1000 mg BID	59
	Clindamycin	150 mg QID	1
	Antianaerobics (Metronidazol, Ornidazol)	500 mg TID	1
**3 days**	Aminopenicillins, Aminopenicillins+Betalactamase Inhibitors	1000 mg BID	12
	Clindamycin	300 mg QID	1
	Clindamycin	150 mg QID	1
	Antianaerobics (Metronidazol, Ornidazol)	500 mg TID	1
**4 days**	Aminopenicillins, Aminopenicillins+Betalactamase Inhibitors	1000 mg BID	2
**5 days**	Aminopenicillins, Aminopenicillins+Betalactamase Inhibitors	1000 mg BID	4
	Total		337

*Abbreviations*: *IM* intramuscular, *IV* intravenous, *BID* twice daily, *TID* 3 times daily, *QID* 4 times daily

**Table 9:** Postoperative antibiotics preferred by the clinicians with regimen and dosage

For	Antibiotic	Dose	***n***
**1 day**	Aminopenicillins, Aminopenicillins+Betalactamase Inhibitors	1000 mg BID	7
**2 days**	Aminopenicillins, Aminopenicillins+Betalactamase Inhibitors	1000 mg BID	7
	Aminopenicillins, Aminopenicillins+Betalactamase Inhibitors	625 mg TID	1
**3 days**	Aminopenicillins, Aminopenicillins+Betalactamase Inhibitors	1000 mg BID	33
	Aminopenicillins, Aminopenicillins+Betalactamase Inhibitors	625 mg TID	3
	Clindamycin	150 mg QID	1
	Spiramycin	3 MIU BID	1
**4 days**	Aminopenicillins, Aminopenicillins+Betalactamase Inhibitors	1000 mg BID	20
	Clindamycin	150 mg QID	1
	Spiramycin	3 MIU BID	1
**5 days**	Aminopenicillins, Aminopenicillins+Betalactamase Inhibitors	1000 mg BID	215
	Aminopenicillins, Aminopenicillins+Betalactamase Inhibitors	625 mg TID	15
	Aminopenicillins, Aminopenicillins+Betalactamase Inhibitors	500 mg TID	3
	Clindamycin	300 mg BID	3
	Clindamycin	150 mg QID	7
	Antianaerobics (Metronidazol, Ornidazol)	500 mg TID	5
	Clarithromycin	500 mg BID	1
	Spiramycin	3 MIU BID	2
	Cephalosporins (IM or IV)	1000 mg BID	1
**6 days**	Aminopenicillins, Aminopenicillins+Betalactamase Inhibitors	1000 mg BID	2
**7 days**	Aminopenicillins, Aminopenicillins+Betalactamase Inhibitors	1000 mg BID	65
	Aminopenicillins, Aminopenicillins+Betalactamase Inhibitors	500 mg TID	1
	Antianaerobics (Metronidazol, Ornidazol)	500 mg TID	1
	Clindamycin (IM or IV)	300 mg BID	1
**8 days**	Aminopenicillins, Aminopenicillins+Betalactamase Inhibitors	1000 mg BID	2
**10 days**	Aminopenicillins, Aminopenicillins+Betalactamase Inhibitors	1000 mg BID	3
	**Total**		402

*Abbreviations*: *IM* intramuscular, *IV* intravenous, *BID* twice daily, *TID* 3 times daily, *QID* 4 times daily

The preoperative and postoperative antibiotic prescription rates of the clinicians according to their practice type (solo private practice, group private practice, and oral dental health center of the state, university clinic) are shown in Tables 10 and 11. There was a statistically significant difference in the preoperative antibiotic prescription rates, but no statistically significant difference in the postoperative case (*p* = 0.002; *p* = 0.182 respectively).

**Table 10:** Preoperative antibiotic prescribing habit by practice type

	Do you prescribe PREoperative antibiotics?			
	Sometimes n (%)	Yes n (%)	No n (%)	*p*
Orodental Health Centre of the State	3 (30)	2 (20)	5 (50)*	**0.002**
University Clinic	36 (65.5)*	9 (16.4)*	10 (18.1)	
Solo Private Practice	90 (38.3)*	96 (40,9)*	49 (20.9)	
Group Private Practice	65 (50.4)	36 (27.9)	28 (21.7)	

*Statistically significant differences

**Table 11:** Postoperative antibiotic prescribing habit by practice type

	Do you prescribe POSTperative antibiotics?			
	Sometimes *n* (%)	Yes *n* (%)	No *n* (%)	*p*
Orodental Health Centre of the State	1 (10)	8 (80)	1 (10)	0.182
University Clinic	5 (9.1)	49 (89.1)	1 (1.8)	
Solo private practice	37 (16.1)	176 (76.5)	17 (7.4)	
Group private practice	17 (13.2)	109 (84.5)	3 (2.3)	

In Table 12, the relationship between pre- and postoperative antibiotic prescription habit of the clinicians (*p* < 0.001) could be seen. While 80% of clinicians prescribing preoperative antibiotics prescribe antibiotics postoperatively; 91.2% of the clinicians do not prescribe antibiotics preoperatively but prescribe antibiotics in the postoperative period.

**Table 12:** Relationship between preoperative and postoperative antibiotic prescribing habit

	Do you prescribe PREoperative antibiotics?			
	Sometimes*	Yes*	No*	*p*
Do you prescribe POSToperative antibiotics?				
Sometimes*	43 (22.3)	12 (8.6)	5 (5.5)	**<0.001**
Yes*	147 (76.2)	112 (80)	83 (91.2)	
No*	3 (1.6)	16 (11.4)	3 (3.3)	

*n (%)

## Discussion

Postoperative infection can be the nightmare of any clinician who is dealing with regenerative and reconstructive oral surgical procedures. The best way to achieve success in daily clinical practice is based on the following evidence-based data. Unfortunately, there is no consensus on some important issues in the current literature and the right perioperative antibiotic regimen in implant surgery is one of them. It is extremely important, especially these days, in which implant dentistry is a part of the routine in our clinical practice, that we are dealing with a serious medico-social event such as antibiotic resistance and with antibiotics’ other adverse effects such as hypersensitivity reactions [11, 13].

It is vital to have evidence-based data to enable our clinical practice to attain a high standard and great success. Success does not mean just the success of the surgical operation or the successful osseointegration of the dental implants. Prescribing antibiotics in a necessary way is also a part of the whole patient care. A study conducted by Marra et al. revealed that between years 1996 and 2013, there was an increase in antibiotic prescribing rates in dental professionals in British Columbia, although there was a decrease in other medical professionals [17]. According to the authors, one of the leading reasons for this situation was increased antibiotic prescribing associated with dental implants and their complications, as well as slow adoption of guidelines which are recommending less prophylactic antibiotic usage for patients with valvular heart diseases and prosthetic joints [17]. The lack of supportive data could induce the clinicians to be pushed into the zones which they found safer than the other options such as prescribing unnecessary antibiotics.

Nowadays, safety does not mean just the patients’ postoperative period without any complications for a clinician, as well as not having any medicolegal issues related to dental procedures including implant surgery. Unfortunately, one of the leading expectations of the patients visiting dental clinics is to be prescribed antibiotics when they have an emergency situation like endodontic emergencies or surgical operations [18]. This situation causes defensive medicine behaviors like prescribing perioperative antibiotics especially after surgical operations like implant placement [19]. Likewise, the problem of malpractice fear could be solved by well-understanding the current scientific data.

As well as the lack of supportive data, the insufficient update mechanism could cause this diversity and different choices in antibiotic prescription related to implant dentistry. A recent study conducted by Kim et al. showed that different interventions such as educational tool guidelines and some restriction tools could induce positive behavioral change in dentists related to implant dentistry, and both simple, and impacted teeth extraction [20]. Also, national organizations are responsible for continuing education of dentists and developing guidelines. However, not only the education of dental professionals will be enough to cope with antibiotic-associated complications in public health. Clinicians should explain to their patients the side effects of antibiotics like hypersensitivity reactions and opportunistic infections. These will work as key instruments in coping with antibiotic resistance, as well as preventing community-associated *Clostridium difficile* infections, and the importance of appropriate antibiotic use [21]. Furthermore, antibiotic stewardship services like in hospitals could provide clinicians better understanding to prescribe antibiotics in an appropriate way with current scientific data [17].

In some cases, educating the clinicians and patients could not be enough to control antibiotic usage. Because of that, controlling the sales of antibiotics like some other drugs by the public healthcare system is beneficial to cope with inappropriate antibiotic usage. In Turkey, antibiotics are sold only on prescription and there are different payment possibilities. Antibiotics are paid by the public healthcare system when the patient is referred from public or university clinics. Furthermore, there is an integrated family medicine system. Every citizen and registered foreigner have their own registered family physician. The patients with an antibiotic prescription from private clinics could get approval from their family physician, in this way the prescribed antibiotics are paid by the public healthcare system. Otherwise, patients are paying by themselves.

Controlling antibiotic prescription by the public healthcare system does not help only appropriate antibiotic prescription or usage. Also, this will help to discover the surveillance in countries and to compare the prescription and resistance rates between countries, years, and different antibiotic families. In recent years, especially aminopenicillins and aminopenicillins with enzyme inhibitors are frequently prescribed antibiotics [17]. Unfortunately, bacterial resistance against this antibiotic family was observed at very high rates in Europe and Central Asia. According to the 2018 data in Surveillance Atlas of Infectious Diseases by European Centre for Disease Prevention and Control, the resistance rates of *Escherichia coli (E.coli)* isolates against amoxicillin ranges between 35.3 and 67.6% showing an increase especially to the southeastern direction and also in Ireland; the resistance rates of *Enterococcus faecium (E.faecium)* isolates against amoxicillin ranges between 62.5 and 97.2%; whose isolates are also showing resistance against vancomycin and high-level gentamicin [22]. In Turkey, the situation is parallel with European countries. According to the annual report of World Health Organization for Central Asian and Eastern European Surveillance of Antimicrobial Resistance in 2019, the resistance rates of *E.coli* isolates against amoxicillin and ampicillin was 77% and amoxicillin with clavulanic acid was 62 %; the resistance rates of *E.faecium* isolates against amoxicillin was 89% [23]. Comparing data in Turkey between 2018 and 2019 showed that in just 1-year resistance against amoxicillin with clavulanic acid has raised from 52 to 62% [23, 24]. As mentioned before, aminopenicillins and aminopenicillins with enzyme inhibitors are frequently prescribed antibiotics combined with dental procedures. Also in this study, the choice of the majority of the clinicians was this antibiotic family. This situation could be interpreted as especially the prescription habits of the dental clinicians are vital when coping with this critical community health crisis.

With the findings of this study, it can be stated that there was no standard antibiotic prescribing habit related to implant dentistry among Turkish clinicians who participated in the questionnaire and our findings are parallel with current literature. In a questionnaire study conducted by Deeb et al., the authors aimed to investigate the antibiotic prescribing habits of the clinicians related to implant surgery [16]. Their findings are parallel with the data of this study. According to their results, they did not have any consensus among clinicians regarding the use of antibiotics related to implant surgery and they observed that the antibiotic regimen chosen by most of the clinicians was not coherent with the current scientific data. Another study conducted with a small sample size by Ireland et al. showed no consensus among clinicians in the United Kingdom (UK) and the authors stated that further studies are needed to develop guidelines regarding antibiotic regimen for implant dentistry in order to prevent unnecessary antibiotic prescription [25]. There are also similar studies from different countries in the literature. Sanchez et al. conducted a cross-sectional questionnaire among dentists and specialists in Netherland and like in this study, they concluded that there was no consensus regarding prescribing antibiotics related to implant surgery and the clinicians were preferring to prescribe antibiotics even if it is not recommended in the guidelines [26].

As mentioned before, the factor that clinicians are not updating themselves with current data could be also one of the reasons why clinicians do not prescribe antibiotics in a proper way. Although systematic reviews and meta-analyses showed that oral administration of 2 g or 3 g of preoperative amoxicillin before 1 h of the operation may be helpful in preventing postoperative infection in peri-implant tissues, only approximately quarter of the respondents has been prescribing preoperative antibiotics for every implant surgery and half of them is practicing this in a right way [5, 8–10]. Half of the respondents who were prescribing preoperative antibiotics in every implant surgery or in specific conditions like the presence of systemic comorbidities were prescribing prophylaxis for more days. Just like in this study, Sanchez et al. also stated that almost half of the clinicians prefer to prescribe preoperative antibiotics when the patient has a systemic condition [26]. These findings are almost parallel with the last consensus report of EAO in which it has been stated that in straightforward cases antibiotic prophylaxis has not shown a beneficial effect; but in complex cases like procedures with grafting or immediate placement of implants and/or patients with systemic comorbidities which are causing disruption in the immune system, antibiotic prophylaxis has a beneficial effect [14]. However, especially for some patients with systemic conditions like valvular heart diseases and prosthetic joints, current guidelines should be followed by the clinician.

Furthermore, the majority of the clinicians were prescribing antibiotics postoperatively and they had almost a consensus for their choice of antibiotics (aminopenicillins and aminopenicillins with beta-lactamase inhibitors with 93.8%). There was a diversity in the duration of antibiotic usage, but more than half of the respondents who were prescribing postoperative antibiotics prefer that their patients took antibiotics for 5 days. A recent systematic review citing 11 RCTs and 3 retrospective studies by Park et al. concluded that postoperative antibiotics should ideally be used in healthy patients with systemic symptoms of infection or in patients with insufficient support from the immune system [27]. Especially when deciding on antibiotic use, the primary factor is the patient’s systemic anamnesis. Decisions should be made on a case-by-case basis. The use of antibiotics should support the appropriate care for patients with complex medical conditions [28, 29]. Parallel with these data, more than half of the respondents (*n* = 220) were prescribing postoperative antibiotics in case of systemic comorbidities, which could cause immunosuppression, but there was a lack of consensus in duration.

In a study conducted by AbuKaraky et al., in which they had investigated the antibiotic prescribing habits of Jordanian clinicians, they investigated the reasons motivating clinicians to prescribe in different procedures related to implant rehabilitation [30]. In the study of AbuKaraky et al., the answers of the respondents showed that the clinicians have more tendency to prescribe more postoperative antibiotics and less preoperative antibiotics when their choice was a surgery with flap elevation. Like in this current study, in multiple implant surgery, clinicians found more safe to antibiotics pre- and postoperatively. Furthermore, the results in AbuKaraky’s study showed that clinicians prefer to prescribe more antibiotics when they were planning to perform implant-related surgeries like bone augmentation or external sinus lift. Because of the design of this current study, these parameters were not comparable, but it could be interpreted overall that clinicians from different countries and educations had shown similar tendencies regarding this subject.

When the data interpreting clinicians’ qualifications and workplace was examined, it was shown that these parameters caused a statistically significant difference for a preoperative antibiotic prescription habit, but not a statistically significant difference for the postoperative one. When the rates of the answers of the respondents were grouped according to their title, the postgraduate students’ choice was to prescribe more preoperative antibiotics and the dentists’ choice was to prescribe fewer preoperative antibiotics; especially in the case of systemic comorbidities (*p* = 0.002). However, the workplace of the clinicians did not have a statistically significant effect on the postoperative antibiotic prescribing habit (*p* = 0.671). Finally, the years of experience both in dentistry and implant dentistry positively affected the antibiotic prescription habit (*p* < 0.001); years in the profession possibly pushed the clinicians to be more or overcautious; or the reason could be the lack of sufficient updating in the current literature.

This study had some important limitations. First of all, the national scope of the study was one of them, but the design of the study had created the possibility to compare our results with similar studies conducted in other countries like Jordan, Netherland, Canada, Ireland, USA, and UK. Furthermore, the rate of the respondents in Turkish society was very low (2.15%). In this study, most of the respondents were frequently performing implant surgery and almost 33.8% of the respondents were specialists for or postgraduate students in implant-related majors. Comparing other similar studies in the scientific literature, the response rate in the study conducted by Deeb et al. in which they investigated perioperative antibiotic prescription habit of the clinicians who were a member of American College of Oral and Maxillofacial Surgeons was 15% (217 of 1436) [16]. Another study conducted by Abukaraky et al., the response rate was 70.4% (176 of 250) and their sample population was Jordanian Dental Implant Group [30]. According to the authors, the reason for the low response rate in this study was that there were not any preselection when creating the sample population. In Turkey, there is not any legal obligation or need to perform implant surgery. Because of that, it had been thought that a general study population could make it possible to understand the antibiotic prescription habit of the Turkish clinicians regarding implant surgery. Further studies would be useful in a more specified sample population. According to the authors, another limitation of this study was that there was not any specification concerning the type of the implant procedure. In the study conducted by Abukaraky et al., they also investigated the clinicians’ antibiotic prescription habits in different types of implant procedures like immediate implant placement in different scenarios; flapless implant placement or implant placement with flap elevation; with sinus lifting procedure or with simultaneous bone augmentation [30]. In this current study, the only specific clinical condition that was asked to the respondents was multiple implant placement. According to the authors, specifying different types and needs of the surgical procedures will affect the clinicians’ choice regarding this subject.

Within the limitations of this study, it could be concluded that in Turkey, there was no consensus on the antibiotic regimen among clinicians dealing with implant dentistry. The lack of sufficient evidence-based data should be supported with well-designed, multi-center, and large-scale RCTs for preoperative and especially for postoperative antibiotic regimens related to implant surgery. Finally, there could be a need for providing recent scientific data to the clinicians, creating some control mechanisms regarding antibiotic prescriptions or antibiotic stewardship like medical hospitals.

## Data Availability

The datasets generated during and/or analyzed during the current study are available from the corresponding author on reasonable request.
